# Adipokines and Inflammation Alter the Interaction Between Rheumatoid Arthritis Synovial Fibroblasts and Endothelial Cells

**DOI:** 10.3389/fimmu.2020.00925

**Published:** 2020-06-02

**Authors:** Rebecca Hasseli, Klaus W. Frommer, Maria Schwarz, Marie-Lisa Hülser, Carina Schreiyäck, Mona Arnold, Magnus Diller, Ingo H. Tarner, Uwe Lange, Joern Pons-Kühnemann, Markus Schönburg, Stefan Rehart, Ulf Müller-Ladner, Elena Neumann

**Affiliations:** ^1^Department of Internal Medicine and Rheumatology, Justus-Liebig-University Giessen, Kerckhoff, Bad Nauheim, Germany; ^2^Medical Statistics, Institute of Medical Informatics, Justus-Liebig University Giessen, Giessen, Germany; ^3^Department of Cardiac Surgery, Kerckhoff-Klinik, Bad Nauheim, Germany; ^4^Department of Orthopedics and Trauma Surgery, Agaplesion Markus Hospital, Frankfurt, Germany

**Keywords:** adipokines, endocrine, fibroblast, rheumatoid arthritis, inflammation, endothelium

## Abstract

**Objective:** The long-distance migration of rheumatoid arthritis synovial fibroblasts (RASFs) in the severe combined immunodeficiency (SCID) mouse model of rheumatoid arthritis (RA) suggests that an interaction between RASFs and endothelial cells (EC) is critical in this process. Our objective was to assess whether immunomodulatory factors such as adipokines and antirheumatic drugs affect the adhesion of RASFs to ECs or the expression of surface molecules.

**Methods:** Primary ECs or human umbilical vein endothelial cell (HUVEC) and primary RASFs were stimulated with adiponectin (10 μg/mL), visfatin (100 ng/mL), and resistin (20 ng/mL) or treated with methotrexate (1.5 and 1,000 μM) and the glucocorticoids prednisolone (1 μM) and dexamethasone (1 μM), respectively. The expression of adhesion molecules was analyzed by real-time polymerase chain reaction. The interaction of both cell types was analyzed under static (cell-to-cell binding assay) and dynamic conditions (flow-adhesion assay).

**Results:** Under static conditions, adipokines increased mostly binding of RASFs to EC (adiponectin: 40%, visfatin: 28%, tumor necrosis factor α: 49%). Under flow conditions, visfatin increased RASF adhesion to HUVEC (e.g., 0.5 dyn/cm^2^: 75.2%). Reduced adhesion of RASFs to E-selectin was observed after treatment with dexamethasone (e.g., 0.9 dyn/cm^2^: −40%). In ECs, tumor necrosis factor α (TNF-α) increased expression of intercellular adhesion molecule 1 (20-fold) and vascular cell adhesion molecule 1 (77-fold), whereas P-selectin was downregulated after stimulation with TNF-α (−6-fold).

**Conclusion:** The adhesion of RASFs to EC was increased by visfatin under static and flow conditions, whereas glucocorticoids were able to decrease adhesion to E-selectin. The process of migration and adhesion of RASFs to ECs could be enhanced by adipokines via adhesion molecules and seems to be targeted by therapeutic intervention with glucocorticoids.

## Key Messages

- Rheumatoid arthritis synovial fibroblast interacts with endothelial cells under static and flow conditions.
- Adipokines, particularly visfatin, might contribute to RA pathogenesis by increasing RASF adhesion to ECs.
- The therapeutic effect of glucocorticoids in RA may partially be explained by reduced RASF/EC adhesion.

## Introduction

Rheumatoid arthritis (RA) is a chronic polyarticular disease, which is characterized by inflammation and joint destruction (1). The RA synovial membrane (synovium), consisting of a lining and sublining layer, is hyperplastic and characterized by increased vascularity and infiltration of immune and stroma cells (1, 2). Rheumatoid arthritis synovial fibroblasts (RASFs) are effector cells and contribute joint inflammation (3, 4). Synovial fibroblasts are able to migrate long distances via the vasculature as previously shown in the severe combined immunodeficiency (SCID) mouse model of RA (4–6), which is mediated by the interaction between RASFs and endothelial cells (ECs) (5). Adhesion molecules and their ligands are involved in the process of migration, which is well-known for immune cell transmigration through vessel walls. Endothelial cells and RASFs are activated by inflammatory factors leading to expression and activation of adhesion molecules, for example, cell adhesion molecules (CAMs) including integrins (7, 8). Upregulation of several adhesion molecules, which mediate adhesion to extracellular matrix (ECM) or cell-to-cell adhesion, is observed in the inflamed RA synovium. For instance, cadherin-11, integrins, and other CAMs are known to be upregulated at sites of inflammation and matrix destruction (7, 9, 10). Cell-to-cell adhesion depends on different adhesion molecules such as selectins that mediate the first steps of adhesion between circulating cells and the endothelium (10). P-selectin and E-selectin are expressed by the endothelium, specifically ECs. Their ligands, such as the E-selectin ligand CD44, Sialyl–Lewis^X^, are expressed by circulating cells. Recently, the role of the cell-cell-adhesion molecule E-selectin during EC and RASF interaction has been shown (11). Osteoarthritis synovial fibroblasts (OASFs) showed lower adhesion properties (11). After the first adhesion steps, further CAMs (10), for example, intercellular adhesion molecule 1 (ICAM-1) and vascular cell adhesion molecule 1 (VCAM-1), are activated and able to interact with other adhesion molecules such as integrins (10).

Both cell-to-cell adhesion and cell-to-ECM adhesion play an important role in inflamed tissues including different compartments of inflamed joints. Of interest, adipose tissue has been found to play a role in inflammatory processes as well (12). Bioactive factors secreted by adipocytes, so-called adipokines (13), have recently been shown to mediate and modulate different inflammatory processes (14). Adipokines induce the secretion of proinflammatory cytokines, such as tumor necrosis factor α (TNF-α), interleukin 6 (IL-6), complement and growth factors, and the upregulation of different adhesion molecules (15, 16). Both RASFs and ECs are affected by adipokines in RA such as adiponectin, visfatin, and resistin (15, 17, 18).

In obesity, diabetes, atherosclerosis, and metabolic syndrome, altered systemic adiponectin levels have been described (19). In RA, increased adiponectin levels were found to be linked with inflammation (20). However, adiponectin seems to have different effects in different diseases. In metabolic and cardiovascular diseases, antidiabetic and antiatherogenic properties were described for adiponectin (21), whereas in RA, high serum adiponectin levels were associated with radiographic damage (22). Adiponectin stimulates the secretion of IL-8, IL-6, matrix metalloprotease 1 (MMP-1), and MMP-13 by RASFs, which contributes to inflammation and joint destruction (23, 24).

Visfatin and resistin are also upregulated in inflammatory processes including RA (15, 18, 25), and serum levels correlate with disease activity (18, 26). Inhibition of visfatin in a mouse model led to reduced arthritis activity (27). Resistin induces the secretion of, for example, TNF-α, IL-6, IL-12, or IL-1β in different cell types (15) including RASFs (14), and intra-articular injection of resistin has been shown to induce synovitis (17).

These findings as well as several other recent reports suggest that adipokines play an important role in inflammation, as well as matrix remodeling and joint damage in RA (15, 28–30). However, the influence of adipokines on the interaction of RASFs and ECs remains unknown.

Glucocorticoids (GCs) are used in rheumatic conditions since decades (31). The treatment of GCs even reduces radiological progression in RA (32). Glucocorticoids bind to a GC receptor (GR), which is localized in the cytoplasm of cells (33) and consists of distinct domains, that is, a binding domain and domains that interact with DNA (33). If GR is activated by binding GCs, GR-GC is able to move to the nucleus and bind to DNA (33–35). Glucocorticoids increase the synthesis of several anti-inflammatory proteins that can suppress inflammation, that is, lipocortin 1 and IL-1 receptor antagonist, which inhibits the proinflammatory effect of phospholipase A2 and IL-1 (33). The transcription of several proinflammatory cytokines is reduced by GCs, including IL-1β, IL-6, and TNF-α (33). The expression of adhesion molecules can be reduced by GCs (33); that is, the expression of adhesion molecules such as ICAM-1 and E-selectin is inhibited at the level of gene transcription (36).

Therefore, in our study, we evaluated the role of selected adipokines (adiponectin, visfatin, resistin) and GCs (prednisolone, dexamethasone) in RASFs–ECs interactions, particularly with regard to adhesion molecules.

## Methods

### Real-Time Polymerase Chain Reaction

RNA was isolated using the RNeasy™ miniprep kit and reverse transcribed (AMV reverse transcriptase; Promega, Walldorf, Germany) using random hexamer primers (Roche Applied Science, Mannheim, Germany). Primer pair (Supplement 1) efficiency was tested using the standard curve method considering 2.00 ± 0.05 as acceptable for experiments. Real-time polymerase chain reaction (PCR) was performed using a LightCycler (Roche Applied Science) with SYBR Green I (Roche Applied Science) as the detection system. Melting curve analysis was used to confirm the specificity of amplification. 18sRNA served as a reference gene. Results were analyzed using the LightCycler software.

### Tissues, Cells, and Cell Culture

Bone fragments, cartilage, and synovium from 14 RA patients (Supplement 7) were obtained during knee replacement surgeries (Department of Orthopedics and Trauma Surgery, Agaplesion Markus-Hospital, Frankfurt, Germany). Patients met the 1987 American College of Rheumatology classification criteria of RA (37). The study was approved by the local ethics committee of the Justus-Liebig-University Giessen. All patients gave written informed consent. Rheumatoid arthritis synovial fibroblasts were isolated and cultured (maximum seven passages) as described (38). After three passages, supplemented Dulbecco modified eagle medium (DMEM) [20% fetal calf serum (FCS), 1 U/mL penicillin/streptomycin, 1 mM HEPES] was replaced by supplemented RPMI (20% FCS, 1 U/mL penicillin/streptomycin, 1 mM HEPES), and RASFs were cultured at 37°C/5% CO_2_ for flow assays. Endothelial cells were isolated from human varicose veins (Departments of Vascular and Cardiac Surgery, Kerckhoff-Klinik, Bad Nauheim, Germany). The vessels were washed twice with phosphate-buffered saline (PBS), and the lumen filled with collagenase H. Ligated vessels were incubated for 1 h at 37°C. Endothelial cell–containing suspension was harvested from the vascular lumen and mixed 1:4 with supplemented DMEM. Cells were centrifuged and resuspended in supplemented DMEM with 0.1 mg/mL EC growth supplement (BD Biosciences, Heidelberg, Germany) and transferred to rat-tail collagen-coated wells. On the next day, adherent cells were washed, and medium changed every 2–3 days for up to three passages to avoid EC dedifferentiation at 37°C/10% CO_2_. Solely EC cultures without fibroblast contamination (vimentin/CD31 immunocytochemical confirmation) were used. At 100% confluence, cells were detached and placed in rat-tail collagen-coated plates. Human umbilical vein endothelial cells (HUVECs) (Promocell, Heidelberg, Germany) were cultured on coated plates with supplemented DMEM with 0.1 mg/mL EC growth supplement for up to two passages.

### Cell-to-Cell Binding Assay

Rheumatoid arthritis synovial fibroblasts were cultured in 12 well-plates and stimulated for 17 h with adiponectin (10 μg/mL; BioVendor, Brno, Czech Republic), visfatin (100 ng/mL; BioVendor), resistin (20 ng/mL; Peprotech, Hamburg, Germany), TNF-α (10 ng/mL; R&D, Bio-Techne Germany, Wiesbaden-Nordenstadt, Germany), prednisolone (1.0 μM; Mibe GmbH, Brehna, Germany), dexamethasone (1.0 μM; Mibe GmbH), or methotrexate (MTX, 1.5 or 1,000 μM; Medac GmbH, Wedel, Germany). The stimulation with TNF-α was used as a positive control as its proinflammatory role, and its ability to increase adhesion molecules in RASFs and ECs is well-known (39–41). The concentrations used for stimulation were based on dose–response analyses with visfatin (25) and adiponectin (42) as performed by our group for previous publications. The concentration for resistin was based on the level that could be detected in synovial fluid (43). The concentration for resistin was based on the level that could be detected in synovial fluid (43). The concentrations of dexamethasone and prednisolone were selected according to publications using both types of GCs to repress inflammation in RASFs (44, 45). The lower MTX concentration corresponds to serum levels as found in RA therapy [MTX (RA)] (46, 47), whereas the higher dose corresponds to serum levels of cancer therapy [MTX (C)] (48, 49). After 17 h, cells were washed with PBS. The stimulation time was chosen based on preliminary experiments that demonstrated an optimal response of stimulation with adipokines after 17 h (data not shown). Viability of RASFs was confirmed by calcein-AM staining for 30 min. Cells were detached with Accutase (Thermo Fisher Scientific GmbH, Dreieich, Germany), and 5 × 10^3^ cells were added to confluent EC layers pretreated for 17 h with TNF-α (R&D) and incubated together for 1 h at 37°C. Supernatants were removed, replaced with serum-free medium (RPMI), and then shaken for 5 min at full speed of the orbital shaker (300 rpm) to remove loosely attached RASFs. This washing step was repeated three times in total using serum-free medium. The medium was removed using a suction device. Attached fluorescent RASFs (on unstained ECs) were quantified in five representative areas each. Confluence of the EC layer was confirmed using bright field microscopy. Results were compared to unstimulated RASFs.

### Flow-Adhesion Assay

**Figure 1:**
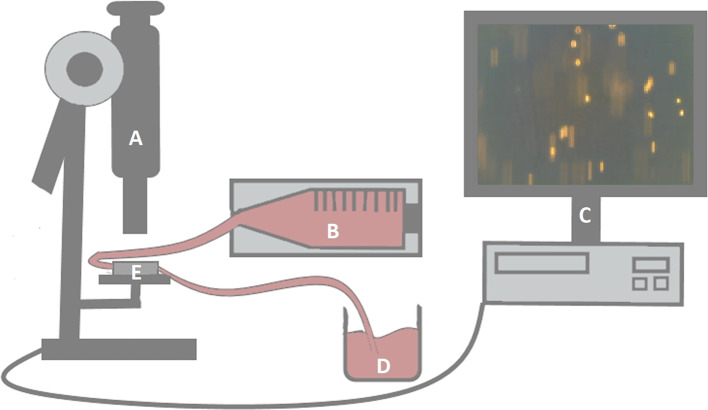
Experimental setup of the flow-adhesion assay. Capillaries **(E)** were monitored microscopically **(A)**. Flow rates of RASF-containing suspensions were regulated by a syringe pump **(B)**. The pump was connected to the capillaries by a tube. Another tube was connected to a collection vessel **(D)** after passing through the capillaries. Synovial fibroblast migration was evaluated by three video sequences per setting **(C)**.

Capillary slides (μ-Slide VI^0.4^, ibiTreat-pretreated; Ibidi, Gräfelfing, Germany) were coated with 30 μL recombinant human E-selectin (1 mg/mL E-selectin/Fc-chimera, 1:20 in PBS) for 1 h at room temperature. Human umbilical vein endothelial cells were added into capillary slides and grown to 100% confluence overnight. Human umbilical vein endothelial cells were activated for 17 h with TNF-α (10 ng/mL; R&D) in DMEM with 0.1 mg/mL EC growth supplement. Rheumatoid arthritis synovial fibroblasts 1.1 × 10^6^ were cultured in supplemented RPMI and stimulated for 17 h with adiponectin, visfatin, or resistin, as well as prednisolone, dexamethasone, or MTX. The stimulation time was chosen based on preliminary experiments that demonstrated an optimal response of stimulation with adipokines after 17 h (data not shown). Results were compared to nonstimulated RASFs. After washing with PBS, RASFs were detached with Accutase, and 0.8 × 10^6^ RASFs were transferred into a syringe pump (Model-100-Series; KD Scientific, Holliston, MA, USA) and connected to the capillary slide. Synovial fibroblast migration through capillaries was monitored microscopically. Cells slowly rolling over the surface and arrested cells were quantified (Figure 1). Means of rolling/arresting cells per visual field were calculated for each recorded sequence (3 × 1 min each). Flow rates of 18.4, 30.5, or 60.5 mL/h, respectively, correspond to shear stress of 0.5, 0.9, or 1.8 dyn/cm^2^, respectively, representing rates detected in postcapillary venules (50, 51).

### Statistics

Data in figures are shown in percentages as box–whisker plots with median, 25th/75th percentile (box), and lowest/highest value (whisker) using SPSS Statistics 24 (IBM, Armonk, New York, United States of America).

In order to analyze adipokine-mediated alteration at different treatments linear mixed models were applied to analyze the repeated measurement design using SPSS Statistics 24 (IBM). Data were log or log2 transformed to reach normal distribution of the residuals, which was verified by Q-Q plots. Estimated marginal means (rhombus) for the fitted models were described together with 95% confidence intervals (CIs). Treatment differences were described by estimated difference and their 95% CIs. All multiple comparisons were Bonferroni adjusted within the analysis of each outcome.

Means, differences, and boundaries of CI were anti–log transformed for the presentation of the results. Issues were regarded as significant for *p* ≤ 0.05. Fold changes of the RT-PCR data were regarded as significant if the 95% CI of log2 transformed –ΔΔct values did not contain 0.

## Results

### Effects of Adipokines and GCs on Adhesion Molecule Gene Expression by RASFs

**Figure 2:**
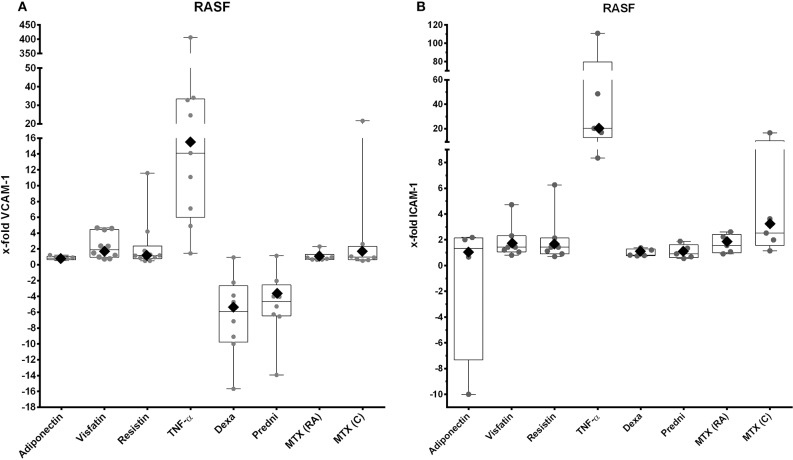
VCAM-1 and ICAM-1 expression by RASFs after stimulation with selected adipokines and therapeutics. Results were compared to non-stimulated controls. **(A)** mRNA expression of VCAM-1 by RASFs after stimulation with adiponectin (*n* = 5), visfatin (*n* = 10), resistin (*n* = 10), TNF-α (*n* = 9), or therapeutics (*n* = 8 each). Adipokines and methotrexate did not have any effect on expression of VCAM-1, whereas TNF-α upregulated expression of VCAM-1 [16.4-fold (rhombus), 95% CI = 4.9–55], but not significantly. Dexamethasone (−5.09-fold, 95% CI = 0.095–0.408) and prednisolone [−3.2-fold (rhombus), 95% CI = 0.14–0.717] downregulated expression of VCAM-1. **(B)** mRNA expression of ICAM-1 by RASFs after stimulation with adiponectin (*n* = 4), visfatin (*n* = 7), resistin (*n* = 7), TNF-α (*n* = 5), and therapeutics (each *n* = 5). Adipokines and therapeutics did not have any effect on expression of ICAM-1. Tumor necrosis factor α significantly upregulated expression of ICAM-1 [20.4-fold (rhombus), 95% CI = 6.07–68].

First, we investigated the influence of adipokines and GCs on the gene expression of selected adhesion molecules. Stimulation with TNF-α increased expression of VCAM-1 (Figure 2A, 16.4-fold, 95% CI = 4.9–55) and ICAM-1 (Figure 2B, 20.3-fold, 95% CI = 6.1–68) significantly. Dexamethasone (Figure 2A, −5.1-fold, 95% CI = 0.095–0.408) and prednisolone (Figure 2A, −3.2-fold, 95% CI = 0.136–0.717) downregulated expression of VCAM-1 significantly. In contrast, none of the adipokines, GCs or MTX, affected the expression of the integrin subunits α2, α4, αν, β1, and β5 on RASFs (data not shown). Expression of cadherin-11 (data not shown), which is overexpressed in RA-synovium (52), as well as VCAM-1 (Figure 2A) and ICAM-1 (Figure 2B), was not significantly changed after stimulation with adipokines or antirheumatic drugs.

### Influence of Adipokines and GCs on Gene Expression of EC Adhesion Molecules

**Figure 3:**
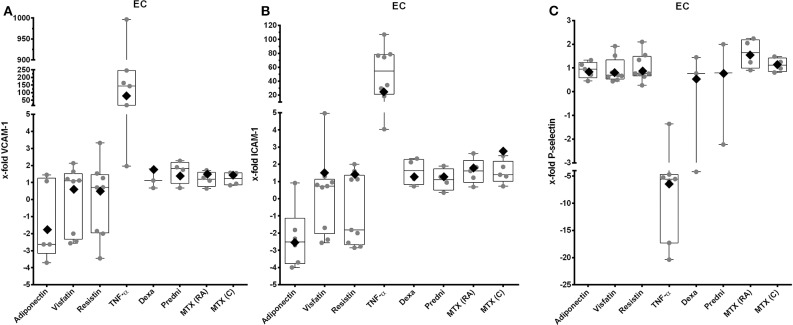
VCAM-1, ICAM-1, and P-selectin expression by EC after stimulation with selected adipokines and therapeutics. Results were compared to non-stimulated controls. **(A)** mRNA expression of VCAM-1 by primary EC after stimulation with adiponectin (*n* = 5), visfatin (*n* = 10), resistin (*n* = 10), TNF-α (*n* = 9), or therapeutics (*n* = 8 each). Adipokines and therapeutics did not have any effect on expression of VCAM-1, whereas TNF-α significantly upregulated expression of VCAM-1 [76.8-fold (rhombus), 95% CI = 11.8–499]. **(B)** mRNA expression of ICAM-1 by primary EC after stimulation with adiponectin (*n* = 6), visfatin (*n* = 9), resistin (*n* = 9), TNF-α (*n* = 8), glucocorticoids (*n* = 4 each), or methotrexate (*n* = 5 each). Adiponectin led to a significant increased expression of ICAM-1 [−2.5-fold (rhombus), 95% CI = 0.26–0.6]. Resistin visfatin and therapeutics did not have any effect on expression of ICAM-1, whereas TNF-α significantly upregulated expression of ICAM-1 [37.02-fold (rhombus), 95% CI = 15–91.4]. **(C)** mRNA expression of P-selectin by primary EC after stimulation with adiponectin (*n* = 5), visfatin (*n* = 8), resistin (*n* = 8), TNF-α (*n* = 7), glucocorticoids (each *n* = 3), or methotrexate (each *n* = 4). Adipokines and therapeutics did not have any effect on expression of P-selectin. Tumor necrosis factor α significantly downregulated expression of P-selectin [−6.3-fold (rhombus), 95% CI = 0.069–0.37].

Tumor necrosis factor α stimulation resulted in a significant overexpression of VCAM-1 (Figure 3A, 77-fold, 95% CI = 11.8–499, Supplement 3) and ICAM-1 (Figure 3B, 20.3-fold, 95% CI = 6.1–68, Supplement 3). Expressions of VCAM-1 and ICAM-1 were not affected by adipokines and antirheumatic drugs (Figures 3A,B), but most of the cell samples showed a decrease of expression of ICAM-1 after stimulation with adiponectin (Figure 3B). Expression of P-selectin was not changed by adipokines and antirheumatic drugs (Figure 3C), whereas stimulation with TNF-α significantly decreased expression (−6.3-fold, 95% CI = 0.069–0.37, Supplement 3).

### RASF Adhesion to ECs Under Static Conditions

**Figure 4:**
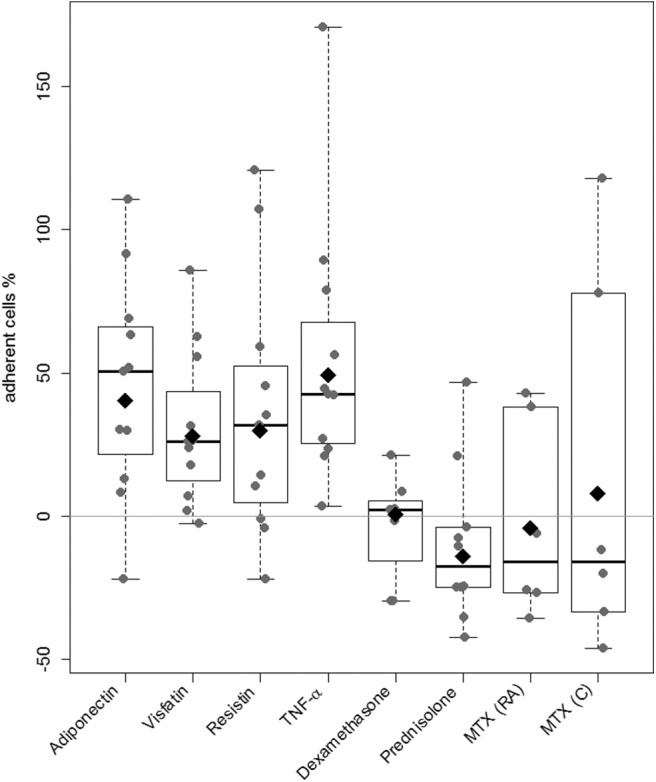
Evaluation of RASFs binding to EC. A cell-to-cell binding assay using primary EC was used to evaluate the effect of the adipokines adiponectin, visfatin, resistin, and TNF-α (*n* = 8 each), glucocorticoids (prednisolone, dexamethasone, *n* = 7 each), and methotrexate (*n* = 6 each dosage). Unstimulated RASFs served as control. The percentage of change in adherent RASFs compared to unstimulated RASFs was calculated. Data in figure are shown in percentages as dot plots with estimated marginal means. Stimulation with selected adipokines increased and prednisolone decreased adhesion to EC in most of the cell samples (adiponectin: 26%, visfatin 19%, resistin 17%, prednisolone: −15%, NS).

Cell-to-cell binding of RASFs to confluent EC layers was increased after stimulation with selected adipokines (adiponectin: 40%, visfatin: 28%, resistin: 30%) compared to nonstimulated control, which was set to 0% (Figure 4). The results for visfatin (*p* = 0.03, Supplement 4) and adiponectin (*p* = 0.048, Supplement 4) were significant. Tumor necrosis factor α, as proinflammatory cytokine, led to a significantly increased adhesion (49%; *p* = 0.004, Supplement 4). Treatment with dexamethasone did not alter adhesion (Figure 4). Although adhesion of both cell types was not changed significantly, most of the cell samples showed a decrease of adhesion in response to prednisolone (8/10) and MTX (C and RA, 4/6 each; Figure 4).

### RASF Adhesion to E-Selectin and HUVECs Under Flow Conditions

**Figure 5:**
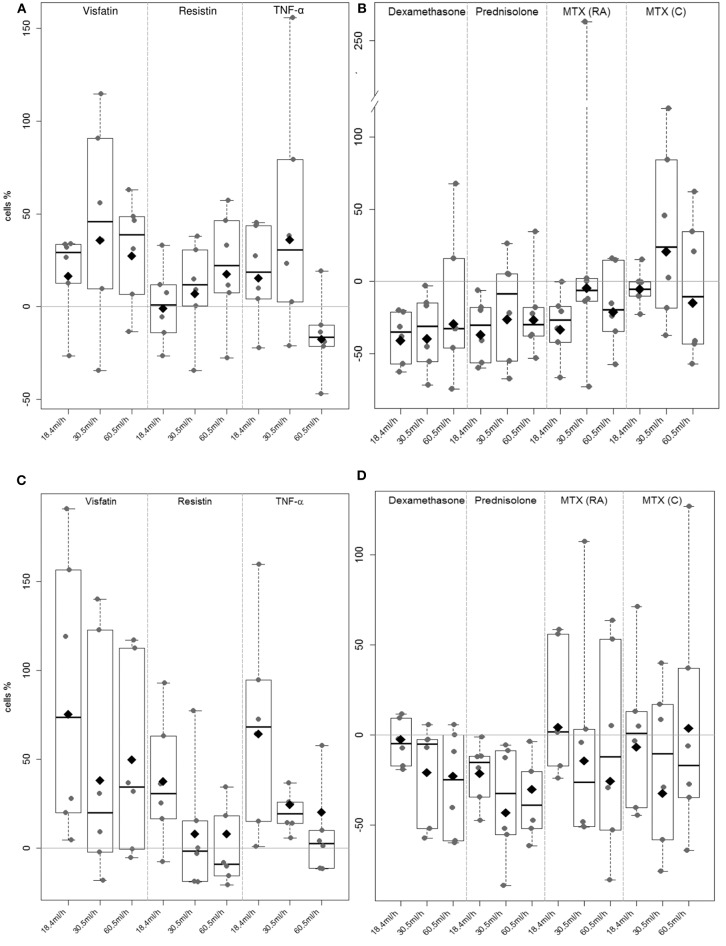
Rheumatoid arthritis synovial fibroblast adhesion to E-selectin and HUVEC under flow conditions. A flow adhesion assay was used to evaluate the effect of the selected adipokines visfatin, resistin, TNF-α (A & C) and therapeutics prednisolone, dexamethasone and methotrexate (B & D) to E-selectin and HUVEC (each *n* = 6). Unstimulated RASF served as control. The percentage of change in adherent RASF compared to unstimulated RASF was calculated. **(A)** Visfatin increased adhesion to E-Selectin in most of the samples (NS). **(B)** Stimulation with dexamethasone significantly (*p* = 0.043) decreased adhesion to E-selectin coated capillaries (8.4 ml/h: −40.9%, 30.5 ml/h: −40%, 60.5 ml/h: −29.7%). **(C)** Significant increase (*p* = 0.002) of adhesion to HUVEC could be observed after stimulation with visfatin (18.4 ml/h: 75.2%, 30.5 ml/h: 37.9%, 60.5 ml/h: 49.8%). **(D)** Stimulation with therapeutics did not reach any significant change in adhesion.

Rheumatoid arthritis synovial fibroblast attachment to E-selectin (Figures 5A,B) and HUVECs (Figures 5C,D) was evaluated in flow-chamber assays. Unstimulated RASFs showed rolling and/or adherence to E-selectin and HUVECs in all settings as shown previously (53). Stimulation with visfatin led to an increased adhesion of RASFs to E-selectin [18.4 mL/h: 16.3%, 30.5 mL/h: 35.7%, 60.5 mL/h: 27.4%; Figure 5A, not statistically significant (NS)]. Resistin (18.4 mL/h: −0.9%, 30.5 mL/h: 6%, 60.5 mL/h: 17%) and TNF-α (18.4 mL/h: 15.4%, 30.5 mL/h: 35.9%, 60.5 mL/h: −17.7%) did not significantly change RASF adhesion to ECs. Treatment with dexamethasone (Figure 5B) reduced interaction of RASFs with E-selectin significantly (8.4 mL/h: −40.9%, 30.5 mL/h: −40%, 60.5 mL/h: −29.7%, Supplement 5). Although prednisolone (18.4 mL/h: −36.9%, 30.5 mL/h: −26.3%, 60.5 mL/h: −26.6%) and MTX (RA) (18.4 mL/h: −33.7%, 30.5 mL/h: −4.5%, 60.5 mL/h: −15.1%) reduced adhesion of RASFs to E-selectin in most patients, the results were not statistically significant (Supplement 5). Methotrexate (C) had no effect on the binding of RASFs to E-selectin.

During cell migration, not only E-selectin is involved in cell interaction and adhesion. Therefore, capillaries were coated with TNF-α-activated HUVECs. Stimulation of RASFs with visfatin (Figure 5C) resulted in significantly (*p* = 0.002, Supplement 6) increased adhesion of RASFs to HUVECs (18.4 mL/h: 75.2%, 30.5 mL/h: 37.9%, 60.5 mL/h: 49.8%). Similar results were observed after stimulation with TNF-α, particularly at lower flow rates (18.4 mL/h: 64.2%, 30.5 mL/h: 24.6%, 60.5 mL/h: 20%, NS). Resistin did not change RASF adhesion significantly (18.4 mL/h: 37.4%, 30.5 mL/h: −0.8%, 60.5 mL/h: 0.8%), although the lowest flow rate was elevated in nearly all samples evaluated. Dexamethasone (18.4 mL/h: −3.6%, 30.5 mL/h: −20.9%, 60.5 mL/h: −22.7%) and prednisolone (18.4 mL/h: −21.4%, 30.5 mL/h: −43.1%, 60.5m l/h: −30.2%) did not change adhesion of both cell types significantly (Figure 5D, Supplement 6), but most of the cell samples showed decreased adhesion.

Methotrexate application (RA and C) increased variation in RASF adhesion in all settings (Figure 5D).

## Discussion

Rheumatoid arthritis synovial fibroblasts play a crucial role in joint damage (38) due to their ability to invade and degrade cartilage and bone and to migrate through the vasculature to distant joints (5). We evaluated in comparison to therapeutic modulation of inflammation whether adipokines have an influence on the interaction between RASFs and ECs by modulating the expression of adhesion molecules on the respective cell surfaces.

In RA, increased angiogenesis takes place because of an imbalance of proangiogenic and anti-angiogenic factors (54). Proinflammatory factors activate the endothelium leading to upregulation of adhesion molecules. Because of these inflammatory processes, hemodynamics is altered, leading to reduced bloodstream velocity. This allows cells, such as lymphocytes and also RASFs, to interact with adhesion molecules on activated ECs. Tumor necrosis factor α induces the expression of adhesion molecules on ECs (55), and TNF-α serum levels are increased in RA (56).

P-selectin is one of the pivotal adhesion molecules in this process. It is located in Weibel–Palade bodies in ECs and translocated to the cell surface following stimulation (57). Recent data suggest an active role of P-selectin in RA (58), and soluble P-selectin is known to be elevated in the serum of RA patients and to correlate with disease activity (58). In our study, stimulation of ECs with selected adipokines did not change the expression of P-selectin in ECs, whereas TNF-α downregulated its expression significantly. Recent data show that P-selectin reaches the maximum of protein expression after 2 h of stimulation with TNF-α (59). After 2 h, a time-dependent downregulation of mRNA and protein expression could be observed (59), which is in line with the observed P-selectin reduction after 17 h in our study. Additionally, flow conditions are required to activate the conformation of P-selectin. Adhesion to P-selectin stops if there is no flow (51), for example, after vessel occlusion.

Similar to the leukocyte adhesion cascade, RASFs were able to interact with ECs via E-selectin under flow conditions, representing one of the first binding partners for leukocytes (5, 10). Similar to P-selectin, flow conditions are required to activate E-selectin (60). Below 18.4 mL/h (0.5 dyn/cm^2^), selectins do not change to their activated conformation. A flow rate of 60.5 mL/h (1.8 dyn/cm^2^) or higher leads to a concentration of cells in the center of the vessel or capillary, and no interaction with the endothelium is possible (50).

In our flow-adhesion assay using E-selectin–coated capillaries, addition of dexamethasone resulted in a significant decrease of RASF adhesion. Thus, dexamethasone might diminish interactions between E-selectin and its ligands, for example, Sialyl–Lewis^X^ (53), as well as CD44, which is also expressed by RASFs (61, 62). Glucocorticoids inhibit the nuclear factor κB pathway (63), potentially influencing adhesion between cells and ECM. In contrast to RASFs, OASFs showed a reduced rolling/adhesion capability to E-selectin in previous experiments (11).

Primary venous ECs and HUVECs showed comparable findings regarding cell numbers in previous experiments (11). Because of the limited availability of ECs, we performed the flow-adhesion assay with HUVECs. In HUVEC-coated capillaries, stimulation with visfatin resulted in a significantly increased adhesion of RASFs to TNF-α-activated ECs. This might be due to induced expression of adhesion molecules on RASFs by visfatin (64). Stimulation with resistin did not show comparable effects in E-selectin- or HUVEC-coated capillaries, although adhesion to HUVECs was increased at the lowest flow rate.

The binding to selectins is followed by the interaction between CAMs and integrins (10). Several integrins of circulating cells, for example, on leukocytes or RASFs, bind to CAMs of ECs, for example, VCAM-1 and ICAM-1. Stimulation of RASFs with adipokines as well as antirheumatic drugs did not change the expression of selected integrins (integrin α2, α4, αν, β1, and β5, data not shown). In contrast, stimulation of ECs with adiponectin reduced ICAM-1. In the context of cardiovascular diseases, adiponectin showed protective effects (21). This could be related to a downregulation of adhesion molecules on ECs. However, because of the chronic inflammatory environment within the synovial tissue, this effect may not be sufficiently strong to reduce influx of cells from the bloodstream into the inflamed joints.

Soluble forms of VCAM-1 and ICAM-1 could be detected in higher concentration in serum of RA patients (65), and both molecules are increased on different cells of the hyperplastic RA synovium (66), including RASFs (41, 67). Ligands of VCAM-1 and ICAM-1 are expressed by leukocytes allowing interactions of both cell types (67, 68). Stimulation of RASFs with TNF-α upregulated the expression of both adhesion molecules significantly. After treatment with dexamethasone and prednisolone, a significant decrease of VCAM-1 mRNA expression was observed. The reduced expression of VCAM-1 by RASFs may diminish adhesion of RASFs to other cells and decrease RASF activity in RA.

The increased adhesion of RASFs to ECs compared to OASF under static conditions was confirmed in cell-to-cell binding assays in previous experiments (11). Under static conditions, stimulation with adiponectin and visfatin increased adhesion of RASFs to ECs significantly, which was comparable to the effect of TNF-α especially after stimulation with adiponectin. Because of the absence of flow conditions, selectins are not involved in cell–cell interactions in this assay. However, the increased RASF–EC binding might be due to the proinflammatory effect of adipokines in RA (69) as expression of the measured adhesion molecules was not altered. Increased adhesion is likely to be promoted by other factors, for example, activation of other adhesion molecules (integrins) and rearrangement on the cell surface (70) besides induced expression of the selected, as well as other adhesion molecules. Stimulation with prednisolone decreased adhesion of RASFs to ECs in most of the cell samples, but results did not reach statistical significance. The decrease of adhesion could be mediated directly or indirectly by altered gene transcription due to GCs. The expression of VCAM-1 was decreased significantly after stimulation with dexamethasone and prednisolone, which may lead to a reduced cell–cell interaction (Figure 4 and Supplement 2).

In RA, RASFs and ECs are located within an inflammatory environment, which contributes to the activation of RASFs and ECs (10, 71). Secreted chemokines and cytokines lead to activation of integrins on the cell surface, as well as induction and activation of other adhesion molecules (10, 51). Our data support the idea that adipokines might play a role in immunomodulation in RA. Especially visfatin enhanced the interaction of RASFs with ECs under flow conditions. *Vice versa*, corticosteroids were able to downregulate VCAM-1 expression in ECs and to reduce adhesion of RASFs to E-selectin under flow conditions. This could explain why corticosteroids are successful in slowing down RA progression. The identification of target molecules responsible for increasing cell adhesion could therefore open new opportunities for RA therapy by targeting these molecules to slow RA progression.

## Conclusion

In this project, we could show that certain adipokines lead to an increase in the adhesion of RASFs to ECs under static and dynamic conditions.

This result suggests that distinct adipokines promote the adhesion of RASFs to the endothelium and thus primarily promote the initial steps of the disease process in the context of the adhesion cascade. The use of dexamethasone and prednisolone resulted in a reduction of RASF adhesion to ECs, especially under flow conditions. This might provide an additional explanation for the protective effect of GCs, which are used in RA therapy.

Interestingly, stimulation with GCs even reduced expression of VCAM-1 by the RASFs, which could affect the binding of leukocytes. This could reduce the recruitment of leukocytes, which could lead to a lower number of immune cells that are maintained in the synovium and contribute to the disease process.

Taken together, the results might open new therapeutic opportunities as, for example, the effect of adipokines could be selectively blocked by antibodies. In addition, the anti-inflammatory effect of TNF-α blockers or the basic drugs (e.g., MTX) could be amplified by adipokine blockers. In contrast, the blockage of a single proinflammatory adipokines is most likely not sufficient to achieve a complete remission of RA, but is worth to consider the combination of the blockade of proinflammatory adipokines and antirheumatic drugs.

## Data Availability Statement

All datasets generated for this study are included in the article/Supplementary Material.

## Ethics Statement

The studies involving human participants were reviewed and approved by Ethics committee of the Justus-Liebig-University Giessen. The patients/participants provided their written informed consent to participate in this study.

## Author Contributions

EN and UM-L designed experiments. RH performed the research, analyzed and interpreted the data, and wrote the manuscript. Synovial fibroblasts from patients with rheumatoid arthritis came from department of orthopedics and trauma surgery, Agaplesion Markus Hospital Frankfurt (SR), and EC came from department of cardiac surgery, Kerckhoff-Klinik Bad Nauheim (MSchö). MSchw, M-LH, CS, MA, and MD contributed to preparation of the research. JP-K contributed to analyze and interpret the data. IT and UL edited the manuscript before submission. All authors read and approved the final manuscript.

## Conflict of Interest

The authors declare that the research was conducted in the absence of any commercial or financial relationships that could be construed as a potential conflict of interest.

## Supplementary Material

Supplement 1 Primer sequences for VCAM-1, ICAM-1, and P-Selectin.

Supplement 2 Evaluation of gene expression after stimulation in RASF. Dexamethasone and prednisolone down-regulated expression of VCAM-1 which was significant. ICAM-1 and VCAM-1 expression was up-regulated after stimulation with TNF-α significantly.

Supplement 3 Evaluation of gene expression after stimulation in EC. TNF-α induced expression of VCAM-1 and ICAM-1 significantly. Adiponectin lead to a significant increased expression of ICAM-1. P-Selectin was significantly down-regulated after stimulation with TNF-α for 17 h.

Supplement 4 Cell-to-Cell binding assay. Stimulation with adiponectin, visfatin, and TNF-α significantly increased adhesion to EC.

Supplement 5 Flow adhesion assay with E-Selectin-coated channels for all velocities. Stimulation with dexamethasone significantly decreased adhesion to E-selectin for all velocities.

Supplement 6 Flow adhesion assay with HUVEC-coated channels for all velocities. Stimulation with visfatin significantly increased adhesion to HUVEC for all velocities.

Supplement 7 Patient characteristics.
