# Supplementary material for: Adipokines and Inflammation Alter the Interaction Between Rheumatoid Arthritis Synovial Fibroblasts and Endothelial Cells
Source: Front Immunol. 2020 Jun 2;11:925. doi: 10.3389/fimmu.2020.00925 (PMC7280538; doi:10.3389/fimmu.2020.00925)
Supplement: Supplement 1 — Primer sequences for VCAM-1, ICAM-1, and P-Selectin. [file Data_Sheet_1.PDF]

Supplement 1: Gene sequences

Gene	Sequence	MgCl2 (mM)	Temperature (°C)
VCAM-1 (forward)<br>VCAM-1 (reverse)	GCAAGAAGGTGGCTCTGTGA<br>AAAGGTGCTGTAGATTCCCATT	3,0	63
ICAM-1 (forward)<br>ICAM-1 (reverse)	TCCTCAGTCAGATACAACAGCATT<br>CTTGAGTCTTGCTCCTTCCTCTT	3,0	63
P-Selectin (forward)<br>P-Selectin (reverse)	CAGGATCTCCCAGTTCCAAA<br>CCAGTTTCCAGTAGCCAAGC	3,0	60
