# Supplementary material for: Adipokines and Inflammation Alter the Interaction Between Rheumatoid Arthritis Synovial Fibroblasts and Endothelial Cells
Source: Front Immunol. 2020 Jun 2;11:925. doi: 10.3389/fimmu.2020.00925 (PMC7280538; doi:10.3389/fimmu.2020.00925)
Supplement: Supplement 2 — Evaluation of gene expression after stimulation in RASF. Dexamethasone and prednisolone down-regulated expression of VCAM-1 which was significant. ICAM-1 and VCAM-1 expression was up-regulated after stimulation with TNF-α significantly. [file Data_Sheet_2.PDF]

Supplement 2: Evaluation of gene expression after stimulation in RASF

Marker	Stimulation	log2 transformed -ΔΔct			anti-log2		
		Mean Difference	95% CI		Fold-change	95% CI	
			Lower Bound	Upper Bound		Lower Bound	Upper Bound
VCAM-1	Adiponektin	-0.280	-0.840	0.279	0.823	0.559	1.213
	Visfatin	0.893	0.136	1.649	1.856	1.099	3.136
	Resistin	0.490	-0.505	1.485	1.404	0.705	2.798
	TNF-α	4.039	2.298	5.781	16.442	4.918	54.972
	Dexamethasone	-2.344	-3.394	-1.293	-5.076	0.095	0.408
	Prednisolone	-1.681	-2.883	-0.479	-3.207	0.136	0.717
	MTX (RA)	-0.098	-0.648	0.451	0.934	0.638	1.367
	MTX (RA)	0.501	-0.972	1.974	1.415	0.510	3.928
ICAM-1	Adiponektin	-0.461	-3.798	2.875	0.726	0.072	7.337
	Visfatin	0.638	-0.147	1.423	1.556	0.903	2.682
	Resistin	0.612	-0.343	1.568	1.529	0.788	2.966
	TNF-α	4.347	2.603	6.091	20.346	6.074	68.154
	Dexamethasone	-0.070	-0.566	0.426	0.953	0.675	1.344
	Prednisolone	-0.043	-0.941	0.855	0.970	0.521	1.808
	MTX (RA)	0.629	-0.184	1.443	1.547	0.880	2.718
	MTX (RA)	1.685	-0.119	3.489	3.216	0.921	11.232
