# Supplementary material for: Adipokines and Inflammation Alter the Interaction Between Rheumatoid Arthritis Synovial Fibroblasts and Endothelial Cells
Source: Front Immunol. 2020 Jun 2;11:925. doi: 10.3389/fimmu.2020.00925 (PMC7280538; doi:10.3389/fimmu.2020.00925)
Supplement: Supplement 3 — Evaluation of gene expression after stimulation in EC. TNF-α induced expression of VCAM-1 and ICAM-1 significantly. Adiponectin lead to a significant increased expression of ICAM-1. P-Selectin was significantly down-regulated after stimulation with TNF-α for 17 h. [file Data_Sheet_3.PDF]

Supplement 3: Evaluation of gene expression after stimulation in EC

Marker	Stimulation	log2 transformed -ΔΔct Mean Difference	log2 transformed -ΔΔct 95% CI Lower Bound	log2 transformed -ΔΔct 95% CI Upper Bound	anti-log2 Fold-change	anti-log2 95% CI Lower Bound	anti-log2 95% CI Upper Bound
VCAM-1	Adiponektin	-0.815	-2.142	0.512	-1.759	0.227	1.426
	Visfatin	-0.168	-0.951	0.615	0.890	0.517	1.532
	Resistin	-0.247	-1.153	0.659	0.843	0.450	1.579
	TNF-α	6.263	3.563	8.963	76.798	11.818	499.062
	Dexamethasone	1.415	-2.712	5.542	2.667	0.153	46.599
	Prednisolone	0.586	-0.661	1.833	1.501	0.632	3.562
	MTX (RA)	0.985	-1.368	3.337	1.979	0.387	10.109
	MTX (RA)	1.015	-1.218	3.248	2.021	0.430	9.504
ICAM-1	Adiponektin	-1.333	-1.923	-0.743	-2.520	0.264	0.597
	Visfatin	-0.368	-1.235	0.499	0.775	0.425	1.414
	Resistin	-0.608	-1.350	0.135	0.656	0.392	1.098
	TNF-α	5.210	3.906	6.514	37.017	14.995	91.383
	Dexamethasone	0.507	-0.761	1.775	1.421	0.590	3.423
	Prednisolone	-0.087	-1.754	1.580	0.941	0.296	2.990
	MTX (RA)	0.534	-0.373	1.441	1.448	0.772	2.716
	MTX (RA)	0.516	-0.316	1.348	1.430	0.803	2.546
P-Selectin	Adiponektin	-0.219	-0.980	0.542	0.859	0.507	1.456
	Visfatin	-0.339	-0.959	0.281	0.791	0.514	1.215
	Resistin	-0.205	-0.971	0.561	0.868	0.510	1.475
	TNF-α	-2.651	-3.854	-1.448	-6.281	0.069	0.367
	Dexamethasone	-0.641	-3.934	2.651	0.641	0.065	6.282
	Prednisolone	-0.165	-2.869	2.538	0.892	0.137	5.807
	MTX (RA)	0.595	-0.379	1.568	1.510	0.769	2.965
	MTX (RA)	0.139	-0.465	0.742	1.101	0.725	1.672
