# Supplementary material for: Adipokines and Inflammation Alter the Interaction Between Rheumatoid Arthritis Synovial Fibroblasts and Endothelial Cells
Source: Front Immunol. 2020 Jun 2;11:925. doi: 10.3389/fimmu.2020.00925 (PMC7280538; doi:10.3389/fimmu.2020.00925)
Supplement: Supplement 4 — Cell-to-Cell binding assay. Stimulation with adiponectin, visfatin, and TNF-α significantly increased adhesion to EC. [file Data_Sheet_4.PDF]

**Supplement 4: Cell-to-Cell binding assay**

| | log2 transformed | | | | anti-log2 | | |
|---|---|---|---|---|---|---|---|
| | | | 95% CI | | | 95% CI | |
| Stimulation | Mean Difference | p-value | Lower Bound | Upper Bound | Change | Lower Bound | Upper Bound |
| Adiponectin | 0.338 | 0.048 | 0.002 | 0.961 | 1.402 | 1.002 | 1.961 |
| Visfatin | 0.247 | 0.030 | 0.015 | 0.615 | 1.280 | 1.015 | 1.615 |
| Resistin | 0.261 | 0.094 | -0.023 | 0.726 | 1.299 | 0.977 | 1.726 |
| TNF-α | 0.401 | 0.004 | 0.096 | 1.033 | 1.493 | 1.096 | 2.033 |
| Dexamethasone | 0.006 | 1.000 | -0.291 | 0.426 | 1.006 | 0.709 | 1.426 |
| Prednisolone | -0.150 | 1.000 | -0.385 | 0.205 | 0.861 | 0.615 | 1.205 |
| MTX (RA) | -0.043 | 1.000 | -0.344 | 0.397 | 0.958 | 0.656 | 1.397 |
| MTX (C) | 0.075 | 1.000 | -0.261 | 0.574 | 1.078 | 1.002 | 1.574 |

All multiple comparisons were Bonferroni adjusted.
