# Supplementary material for: Adipokines and Inflammation Alter the Interaction Between Rheumatoid Arthritis Synovial Fibroblasts and Endothelial Cells
Source: Front Immunol. 2020 Jun 2;11:925. doi: 10.3389/fimmu.2020.00925 (PMC7280538; doi:10.3389/fimmu.2020.00925)
Supplement: Supplement 5 — Flow adhesion assay with E-Selectin-coated channels for all velocities. Stimulation with dexamethasone significantly decreased adhesion to E-selectin for all velocities. [file Data_Sheet_5.PDF]

Supplement 5: Flow adhesion assay with E-Selectin-coated channels for all velocities

	log2 transformed				anti-log2		
			95% CI			95% CI	
Stimulation	Mean Difference (I-J)	Significance (Bonferroni adjusted)	Lower Bound	Upper Bound	Mean Difference	Lower Bound	Upper Bound
Visfatin	0.233	0.377	-0.095	0.560	1.262	0.909	1.751
Resistin	0.073	1.000	-0.332	0.478	1.076	0.717	1.614
TNF-α	0.085	1.000	-0.355	0.526	1.089	0.701	1.691
Dexamethasone	-0.462	0.043	-0.916	-0.008	0.630	0.400	0.992
Prednisolone	-0.358	0.223	-0.810	0.094	0.699	0.445	1.098
MTX (RA)	-0.232	1.000	-0.665	0.201	0.793	0.514	1.222
MTX (RA)	-0.011	1.000	-0.399	0.377	0.989	0.671	1.458

Flow adhesion assay with E-Selectin-coated channels for the respective velocities

		log2 transformed				anti-log2		
				95% CI			95% CI	
Flow	Stimulation	Mean Difference	Significance (Bonferroni adjusted)	Lower Bound	Upper Bound	Mean Difference	Lower Bound	Upper Bound
18.4 ml/h	Visfatin	0.151	1.000	-0.418	0.719	1.163	0.658	2.052
	Resistin	-0.009	1.000	-0.716	0.698	0.991	0.489	2.010
	TNF-α	0.143	1.000	-0.631	0.918	1.154	0.532	2.505
	Dexamethasone	-0.526	0.548	-1.338	0.286	0.591	0.262	1.331
	Prednisolone	-0.460	0.925	-1.293	0.373	0.631	0.274	1.453
	MTX (RA)	-0.410	1.000	-1.256	0.435	0.663	0.285	1.545
	MTX (RA)	-0.057	1.000	-0.910	0.796	0.945	0.403	2.216
30.5 ml/h	Visfatin	0.305	1.000	-0.263	0.874	1.357	0.768	2.395
	Resistin	0.066	1.000	-0.641	0.773	1.068	0.527	2.165
	TNF-α	0.307	1.000	-0.468	1.081	1.359	0.626	2.949
	Dexamethasone	-0.509	0.618	-1.321	0.303	0.601	0.267	1.354
	Prednisolone	-0.306	1.000	-1.139	0.528	0.737	0.320	1.695
	MTX (RA)	-0.046	1.000	-0.891	0.800	0.955	0.410	2.225
	MTX (RA)	0.188	1.000	-0.665	1.041	1.207	0.514	2.832
60.5 ml/h	Visfatin	0.242	1.000	-0.326	0.811	1.274	0.722	2.250
	Resistin	0.162	1.000	-0.545	0.869	1.176	0.580	2.385
	TNF-α	-0.194	1.000	-0.969	0.581	0.823	0.379	1.787
	Dexamethasone	-0.352	1.000	-1.164	0.460	0.703	0.312	1.584
	Prednisolone	-0.309	1.000	-1.142	0.524	0.734	0.319	1.689
	MTX (RA)	-0.240	1.000	-1.086	0.605	0.786	0.338	1.832
	MTX (RA)	-0.163	1.000	-1.016	0.690	0.849	0.362	1.993
