# Supplementary material for: Adipokines and Inflammation Alter the Interaction Between Rheumatoid Arthritis Synovial Fibroblasts and Endothelial Cells
Source: Front Immunol. 2020 Jun 2;11:925. doi: 10.3389/fimmu.2020.00925 (PMC7280538; doi:10.3389/fimmu.2020.00925)
Supplement: Supplement 6 — Flow adhesion assay with HUVEC-coated channels for all velocities. Stimulation with visfatin significantly increased adhesion to HUVEC for all velocities. [file Data_Sheet_6.PDF]

Supplement 6: Flow adhesion assay with EC-coated channels for all velocities

	log2 transformed				anti-log2		
			95% CI			95% CI	
Stimulation	Mean Difference (I-J)	Significance (Bonferroni adjusted)	Lower Bound	Upper Bound	Mean Difference	Lower Bound	Upper Bound
Visfatin	0.429	0.002	0.118	0.739	1.536	1.125	2.095
Resistin	0.158	1.000	-0.238	0.554	1.171	0.788	1.740
TNF-α	0.300	0.447	-0.139	0.739	1.350	0.870	2.093
Dexamethasone	-0.172	1.000	-0.628	0.283	0.842	0.534	1.328
Prednisolone	-0.388	0.142	-0.840	0.063	0.678	0.432	1.065
MTX (RA)	-0.135	1.000	-0.567	0.297	0.873	0.567	1.345
MTX (RA)	-0.142	1.000	-0.539	0.256	0.868	0.583	1.292

Flow adhesion assay with EC-coated channels for the respective velocities

		log2 transformed				anti-log2		
				95% CI			95% CI	
Flow	Stimulation	Mean Difference	Significance (Bonferroni adjusted)	Lower Bound	Upper Bound	Mean Difference	Lower Bound	Upper Bound
18.4 ml/h	Visfatin	0.561	0.038	0.019	1.103	1.752	1.019	3.012
	Resistin	0.318	1.000	-0.382	1.017	1.374	0.683	2.765
	TNF-α	0.496	0.615	-0.293	1.284	1.642	0.746	3.613
	Dexamethasone	-0.026	1.000	-0.868	0.816	0.974	0.420	2.262
	Prednisolone	-0.241	1.000	-1.113	0.631	0.786	0.329	1.880
	MTX (RA)	0.043	1.000	-0.851	0.937	1.044	0.427	2.552
	MTX (RA)	-0.070	1.000	-0.984	0.844	0.932	0.374	2.325
30.5 ml/h	Visfatin	0.322	0.745	-0.220	0.863	1.379	0.802	2.371
	Resistin	0.077	1.000	-0.622	0.777	1.080	0.537	2.175
	TNF-α	0.220	1.000	-0.569	1.009	1.246	0.566	2.742
	Dexamethasone	-0.234	1.000	-1.076	0.608	0.791	0.341	1.838
	Prednisolone	-0.564	0.549	-1.437	0.308	0.569	0.238	1.360
	MTX (RA)	-0.153	1.000	-1.047	0.740	0.858	0.351	2.097
	MTX (RA)	-0.392	1.000	-1.307	0.522	0.675	0.271	1.685
60.5 ml/h	Visfatin	0.404	0.304	-0.138	0.946	1.498	0.871	2.574
	Resistin	0.078	1.000	-0.621	0.778	1.081	0.537	2.177
	TNF-α	0.184	1.000	-0.605	0.972	1.201	0.546	2.643
	Dexamethasone	-0.257	1.000	-1.099	0.585	0.773	0.333	1.796
	Prednisolone	-0.359	1.000	-1.231	0.513	0.698	0.292	1.671
	MTX (RA)	-0.296	1.000	-1.190	0.598	0.744	0.304	1.818
	MTX (RA)	0.038	1.000	-0.883	0.959	1.038	0.413	2.608
