# Supplementary material for: Adipokines and Inflammation Alter the Interaction Between Rheumatoid Arthritis Synovial Fibroblasts and Endothelial Cells
Source: Front Immunol. 2020 Jun 2;11:925. doi: 10.3389/fimmu.2020.00925 (PMC7280538; doi:10.3389/fimmu.2020.00925)
Supplement: Supplement 7 — Patient characteristics. [file Data_Sheet_7.PDF]

Supplement 7: Characteristics of rheumatoid arthritis patients

Number of Patients	14
Gender	11 ♀; 3♂
Age (years)	66 (25-88)
Disease Duration (years)	13 (2-45)
DMARD at time of joint replacement	Methotrexate: 9<br>TNF-alpha-Inhibitor & Methotrexate: 3
Prednisolone	n=8<br>6,6 mg/d (2-20)
